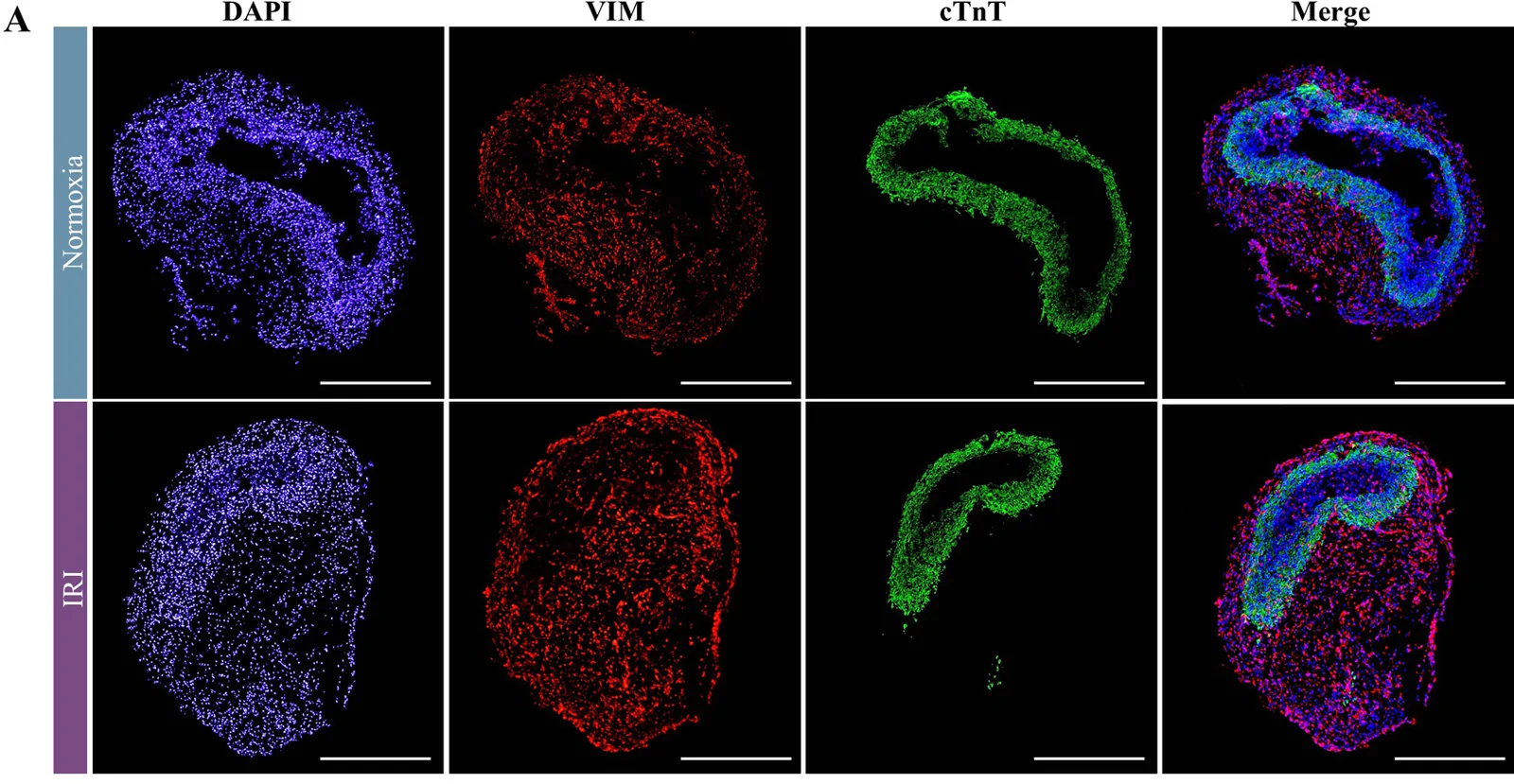# An Integrated Cardiac Microtissue Proteome Map Extends Therapeutic Remodeling by Nanovesicles

**DOI:** 10.1016/j.mcpro.2026.101643

**Published:** 2026-08-20

**Authors:** Jonathan Lozano, Jarmon G. Lees, Jonathon Cross, Taha Aghajanzadeh, Haoyun Fang, Anne M. Kong, Ren J. Phang, Alice Pébay, Alin Rai, Shiang Y. Lim, David W. Greening

**Affiliations:** 1 Molecular Proteomics, Baker Heart and Diabetes Institute, Melbourne, Victoria, Australia; 2 Baker Department of Cardiovascular Research, Translation and Implementation La Trobe University, Melbourne, Victoria, Australia; 3 O'Brien Institute Department, St Vincent's Institute of Medical Research, Victoria, Australia; 4 Department of Surgery and Medicine, University of Melbourne, Melbourne, Victoria, Australia; 5 Drug Discovery Biology, Monash Institute of Pharmaceutical Sciences, Monash University, Victoria, Australia; 6 Baker Department of Cardiometabolic Health, University of Melbourne, Melbourne, Victoria, Australia; 7 Department of Surgery, Melbourne Medical School, University of Melbourne, Melbourne, Victoria, Australia; 8 Department of Anatomy and Physiology, School of Biomedical Sciences, University of Melbourne, Melbourne, Victoria, Australia; 9 National Heart Research Institute Singapore, National Heart Centre, Singapore, Singapore

**Keywords:** cardiac microtissue, human pluripotent stem cells, endothelial, cardiomyocyte, fibroblast, nanovesicles, proteome remodeling

## Abstract

Human cardiac microtissues are a promising model to study cardiac biology and disease, but their application is constrained by therapeutic remodeling strategies and limited knowledge of their functional protein expression profiles. Here, we define the use of human cardiac microtissue (hCMT) model generated by assembling induced pluripotent stem cell-derived endothelial cells, cardiac fibroblasts, and cardiomyocytes (CMs) to model ischemia-reperfusion injury (IRI) through a model of hypoxia and reoxygenation and nanovesicle (NV)-mediated functional remodeling. Engineered NVs, generated directly from human stem cells, have been shown to influence cardiac tissue and cell repair, and provide a platform for scalable and reproducible cell free-mediated therapy. We show the functional regulation of the hCMT model and define that administration of NVs (from human induced pluripotent stem cell origin) during reoxygenation significantly increase CM survival and preserve contractility function (contractile duration, relaxation time, and relaxation:contraction velocity). We establish NV uptake and transfer with target cells from the hCMT model. Quantitative proteomics was applied to decipher the cell proteome dynamics and molecular mechanisms of IRI in our *in vitro* model following NV treatment, linked with networks associated with cell survival, energy production, and stress response regulation. Notably, cell type-specific enrichment analysis revealed that NVs drive distinct proteomic remodeling based on their cell origin, where CERA NVs selectively upregulate cytoprotective and structural networks (such as HSP70, MYH6, and XIRP1) within parenchymal CMs, whereas CL2 NVs predominantly suppress non-myocyte activation and extracellular matrix remodeling factors within the endothelial and fibroblast compartments. Our findings provide an advanced human stem cell-based platform to understand underlying mechanisms of IRI and assess cell-free therapeutic cardioprotective strategies.

## Highlights

• Human cardiac microtissues recapitulate features of ischemia–reperfusion injury.
• Proteomics reveals 293 proteins remodeled by cardiac injury.
• iPSC nanovesicles restore survival and contractile function following injury.
• Nanovesicles induce cell type–specific, pro-reparative proteome remodeling.

## Graphical Abstract

**Figure:**
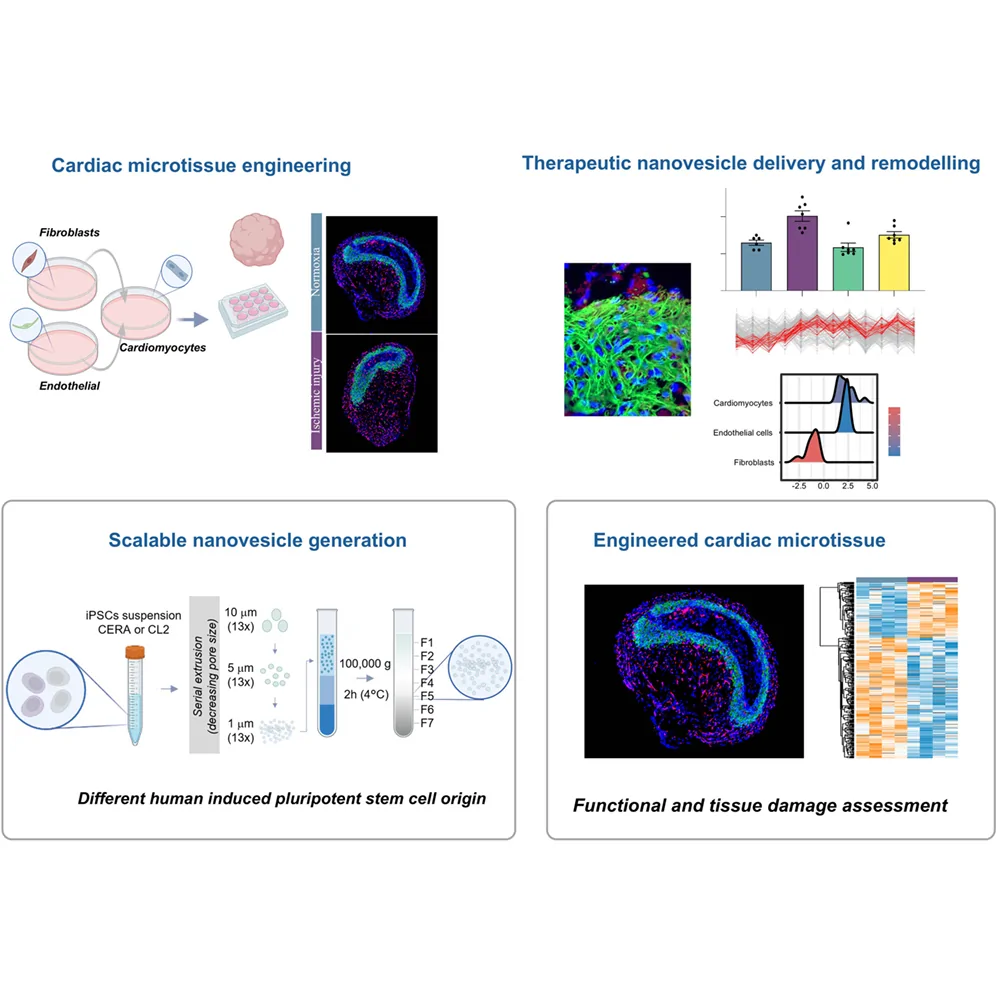

## In Brief

Advanced human stem cell-based platform provides a cardiac microtissue model to understand nanovesicle-based function and proteome remodeling, with potential applications for disease modeling and therapeutic intervention.

The human heart is an extraordinary organ composed of a diverse array of cell types, including abundant cardiomyocytes (CMs), fibroblasts, and endothelial cells, that function collaboratively to maintain its rhythmic contractile activity and systemic blood circulation (1). This orchestrated interplay is critical for adapting to both physiological demands and pathological challenges (2). However, when the coronary arteries are occluded, as in the case of myocardial infarction (MI), the resulting ischemia leads to CM death, triggering a cascade of inflammatory responses and fibrotic remodeling that irreversibly compromise heart function (3). Despite advances in rapid reperfusion therapies that have emerged, many patients still develop long-term complications, including adverse ventricular remodeling and heart failure, as the regeneration of lost myocardium remains extremely limited in the adult heart (4, 5). Importantly, reperfusion injury following ischemia (due to the sudden tissue reoxygenation, termed IRI) exacerbates the initial injury and induces further cell death (6, 7, 8).

The difficulty of studying and modeling the human heart to evaluate and develop advanced therapeutics underscores the need for standardized, representative *in vitro* models of human cardiac biology. Given that multiple cell types are involved in MI-induced acute cardiac injury and together they build a signaling network which drives tissue remodeling, *in vitro* modeling warrants the mimicking of cellularity and integration to be physiologically representative (9). This versatile and dynamic network cannot be recapitulated entirely in 2D monolayer culture. In this regard, scaffold-free tissue engineering approaches offer unique opportunities for developing 3D models of the heart muscle in a microtissue (MT) structure (10, 11). In this format, CMs can be seeded alone or in combination with other cardiac cell types, allowing cell aggregation and subsequent tissue formation, and mimicking the native physiological state (12, 13, 14, 15). Here, we defined the use of human cardiac microtissue (hCMT) model from endothelial, fibroblast, and CM cells from induced pluripotent stem cell (iPSC) origin. These multicellular cardiac MTs autonomously contract and exhibit key features of CMs, cardiac fibroblasts, and endothelial cells, including organized sarcomeres, calcium handling, and electrical activity, an intrinsic complexity to model ischemic reperfusion injury (IRI) (12, 16).

To limit the acute injury response following MI damage, regenerative strategies have garnered significant preclinical and clinical interest, including cell-based and cell-free therapies (17). Although a variety of cell-based approaches have shown potential in preclinical studies, challenges such as poor cell retention, immune rejection, and the risk of arrhythmogenesis have hindered their current clinical feasibility (18, 19). As a cell-free therapeutic strategy, nanosized cell-derived extracellular vesicles (EVs) have emerged as promising cell-free therapeutic strategy for cardiac repair (20, 21) due to their biophysical features in cardiac protection, feasibility in modification to enhance their bioactivity and extend circulation time, and derived from different stem cell sources (21, 22, 23). Furthermore, these stem cell-derived EVs can induce potent reparative effects to tissues and cells (24, 25, 26, 27, 28), can be targeted for their delivery and retention to the heart (29), and recently EVs from regenerating tissue have been shown to induce protective and regenerative mechanisms directly to the heart (20). As EV-like mimetics, NVs are sourced directly from stem cells, and overcome challenges in using natural EVs in their scalable generation, high yield, rapid and reproducible generation (30, 31), customizable engineering for bioactivity (32) and targeted delivery, while still retaining natural cell-like properties such as low immunogenicity and relevant surface proteins for function, making them superior for scalable drug delivery (33). We have previously reported stem cell-derived NVs for cell-mediated uptake and their functional capacity (31, 32) and to promote CM survival, endothelial cell angiogenesis, and attenuate primary cell fibrotic response (34).

We have previously described the generation of 3D cardiac aggregates (MT) (12) using human iPSC-derived cardiac cell types (16, 35). Integration of multiple cardiac cell types in these 3D cultures has been shown to promote the expression of CM maturity markers and better recapitulate *in vivo* microenvironment (36). This multi-cell cardiac system exhibits spontaneous contraction, measurable extracellular field potentials, dynamic calcium cycling, and respond appropriately to chronotropic agents such as isoprenaline and carbachol (12, 16, 35). These features make hCMTs a reliable platform for modeling complex cardiac pathologies, including IRI (16, 37), diabetes-associated metabolic stress (35, 38), and doxorubicin-induced cardiotoxicity (12). Furthermore, the hCMTs have proven effective in evaluating therapeutic interventions, such as metformin for diabetes-associated metabolic stress (38) and mesenchymal stromal cell-based therapies for IRI (37), highlighting their translational relevance for disease modeling and drug testing. These characteristics highlight the advantages of cardiac MTs over traditional 2D cultures. Here, we aimed to employ a hCMT model featuring multiple and abundant cell types of the human heart (endothelial, fibroblast, and CM cells) with cardiac identity to model IRI using hypoxia and reoxygenation and assess NV-mediated functional and proteome remodeling. Furthermore, we assess cell uptake of NVs by the hCMT model using lipophilic dye labeled NVs and assess their cell type interaction/uptake in the context of hypoxia and reoxygenation. This work shows the potential of using NVs to trigger protective and regenerative mechanisms in the context of IRI on human cardiac MTs.

## Experimental Procedures

### Experimental Design and Statistical Rationale

In this study, we aimed to employ a human cardiac MT model with cardiac identity to model IRI and assess proteome and functional remodeling highlighting translational relevance in cardiac regenerative therapeutics. For iPSC source, we used human iPS-Foreskin-2 (CL2) cells and CERA007c6 (CERA) iPSC line. Human cardiac MT was generated from defined populations of human iPSC-derived CMs, endothelial cells, and cardiac fibroblasts, in cell-specific proportion. For functional assessment including cardiac troponin T (cTnT) to assess CM cell survival; normoxia n = 6, IRI n = 7 (biological replicates), with cardiac MT proteomics n = 4 per group (Normoxia UT, IRI UT, CL2 NVs, and CERA NVs) (biological replicate). Hierarchical cluster analysis of normoxia and IRI MT samples based on distinct protein expression profiles (ANOVA, fold change ± 2.0). For each biological replicate (n = 4) across each group, single-shot data-dependent acquisition (DDA)-MS were obtained (16 MS files).

For proteomic experiment data, we conducted Pearson correlation analysis, hierarchical clustering using Euclidian distance and average linkage clustering, in addition to nonlinear iterative partial least squares principal component analysis (PCA) to evaluate the variations between replicates, overall data quality and the effect of missing value imputations. We performed one-way ANOVA and student’s *t* test with Benjamini–Hochberg for multiple-testing correction. To evaluate cell-type representation, gene set enrichment analysis (GSEA) was performed for differential proteome analyses of each NV group relative to IR (clusterProfiler in R) and statistical significance adjusted for multiple testing using the Benjamini-Hochberg false discovery rate (FDR); adjusted *p*-value (Benjamini–Hochberg-corrected) < 0.05 as indicated and |log2 fold-change|>1 deemed statistically significant.

### Cell Culture

#### Human iPSCs

Human iPS-Foreskin-2 (CL2) cell line (39), kindly provided by James A. Thomson (University of Wisconsin), and CERA007c6 (CERA) (40) iPSC line were maintained on vitronectin-coated plates in TeSR-E8 medium (Stem Cell Technologies) according to the manufacturer’s protocol. Briefly, cells were cultured until confluent, then detached using an enzyme-free dissociation reagent (ReLeSR, Stem Cell Technologies). The resulting cell aggregates were reseeded onto new vitronectin-coated plates for further expansion. Medium was replaced every 2 to 3 days. Brightfield images were obtained using an inverted microscope (Olympus IX71) at 40× magnification.

### CM Differentiation from iPSCs

CMs were derived from human iPSCs as previously described with modifications (37, 41, 42). Briefly, human iPSCs were seeded onto human embryonic stem cell qualified-Matrigel (Corning) coated plates at a density of 1.25 × 10^5^ cells/cm^2^ in TeSR-E8 medium supplemented with 10 μM Y−27632 (Abcam). After 48 h (day 0), medium was replaced with RPMI-1640 basal medium (Thermo Fisher Scientific) containing B-27 without insulin supplement (Thermo Fisher Scientific), growth factor reduced Matrigel (1:60 dilution) and 10 μM (for CL2 iPSCs) or 6 μM (CERA iPSCs) CHIR99021 (Cayman Chemical). At day 1, medium was replaced with RPMI 1640 basal medium containing B-27 without insulin supplement. At day 2, medium was changed to RPMI 1640 basal medium containing B-27 without insulin supplement and 5 μM IWP-2 (Sigma-Aldrich) for 72 h. From day 5, cells were cultured in RPMI 1640 basal medium containing B-27 supplement (Thermo Fisher Scientific) and 200 μg/ml L-ascorbic acid 2-phosphate sesquimagnesium salt hydrate (Sigma-Aldrich) (CM medium, v), with CMm changed every 2 to 3 days. At day 12, differentiated CMs were dissociated into single cells and split 1:4 onto growth factor reduced Matrigel-coated plates in DMEM/F-12 GlutaMAX medium supplemented with 20% FBS (Sigma-Aldrich), 0.1 mM 2-mercaptoethanol, 0.1 mM nonessential amino acids, 50 U/ml penicillin/streptomycin and 10 μM Y−27632. At day 13, medium was changed to CMm. From days 14 to 19, CMs were enriched to >95% cTn T positive cells by culturing in glucose-free DMEM medium (Thermo Fisher Scientific) supplemented with 4 mM lactate (Sigma-Aldrich).

### Endothelial Cell Differentiation from iPSCs

Human iPSCs were differentiated into CD31+ endothelial cells according to a previously published method (16, 37, 43). For endothelial differentiation, iPSCs were dissociated into single cells and seeded onto growth factor reduced Matrigel-coated plates at a density of 1 × 10^5^ cells/cm^2^ in TeSR-E8 medium supplemented with 10 μM Y−27632. After 24 h, referred to as day 0, medium was replaced with DMEM/F12 GlutaMAX medium (Thermo Fisher Scientific) containing N-2 supplement (Thermo Fisher Scientific), B27 supplement, 8 μM CHIR99021 and 25 ng/ml BMP4 (STEMCELL Technologies) for 3 days. Medium was then replaced with StemPro-34 SFM complete medium (Thermo Fisher Scientific) supplemented with 200 ng/ml VEGF-165 (Peprotech) and 2 μM forskolin (Sigma-Aldrich) for 3 days. At day 6, CD31 positive cells were sorted by FACS using a CD31-conjugated antibody (BD Pharmingen), and expanded on human fibronectin (Merck) coated plates and cultured in EGM2-MV medium (Lonza) supplemented with 50 ng/ml VEGF-165.

### Cardiac Fibroblast Differentiation from iPSCs

Human iPSCs were differentiated into cardiac fibroblasts according to a previously published method (16, 36, 37). iPSCs were dissociated into single cells and seeded onto Matrigel-coated plates at a density of 2.5 × 10^4^ cells/cm^2^ in TeSR-E8 medium supplemented with 10 μM Y−27632. After 24 h, referred to as day 0, medium was replaced with STEMdiff APEL medium (STEMCELL Technologies) containing 20 ng/ml BMP4, 20 ng/ml Activin A (Peprotech), and 1.5 μM CHIR99021 for 3 days. On day 3, medium was replaced with STEMdiff APEL medium containing 30 ng/ml BMP4, 1 μM retinoic acid (Sigma-Aldrich), and 5 μM IWP-2 (Sigma-Aldrich), and then replaced again on day 6 but without IWP-2. On day 9, cells were dissociated and reseeded at 1.5 × 10^4^ cells/cm^2^ on human fibronectin in STEMdiff APEL medium containing 10 μM SB431542 (Tocris Bioscience) and 10 μM Y−27632. On day 13, epicardial cells were dissociated and reseeded at 2.5 × 10^4^ cells/cm^2^ on 0.1% porcine gelatine-coated plates and cultured in STEMdiff APEL medium containing 10 ng/ml bFGF (Peprotech) and 10 μM Y−27632 and medium was changed every 2 days without Y−27632. On day 20, the medium was changed to FGM-3 (Lonza) and replaced every 2 to 3 days until confluent.

### Engineered Cardiac MTs

To construct the cardiac MTs, as previously described (12, 16, 38), enriched day-19 iPSC-derived CMs (1.2 × 10^5^ cells/well) were seeded onto Matrigel-coated 48-well NuncTM UpCell plates in DMEM/F-12 GlutaMAX medium supplemented with 20% fetal bovine serum (Sigma-Aldrich), 0.1 mM 2-mercaptoethanol, 0.1 mM nonessential amino acids, 50 U/ml penicillin/streptomycin and 10 μM Y−27632. After 24 h, iPSC-derived endothelial cells (7 × 10^4^ cells/well) and iPSC-derived cardiac fibroblasts (1 × 10^4^ cells/well) were then seeded onto the iPSC-CMs and cultured in cardiac MT medium consisting of a mixture of CMm, EGM2-MV medium, and FGM-3 medium (at 1:1:1 ratio) supplemented with 50 ng/ml VEGF-165. After 24 h, the UpCell plates were brought to RT, and the detached cell sheet was transferred to an ultralow attachment plate (Sigma-Aldrich) containing cardiac MT medium for 24 h. The resulting spheroids were then embedded in 15 μl of growth factor reduced Matrigel and cultured in cardiac MT medium, designated as day 0. Cardiac MTs were maintained in culture in a humidified CO2 incubator on an orbital shaker at 100 rpm and medium was changed every 2 to 3 days.

### Immunofluorescent Staining of Engineered Cardiac MT

Engineered cardiac MTs were fixed in 10% neutral buffered formalin for 1 h at RT and then dehydrated in 20% sucrose solution for 24 h. Dehydrated samples were embedded in Optimal Cutting Temperature compound (Sakura Finetek) and cryosections (6 μm thick) were treated with 0.2% Triton X-100 permeabilization buffer and Protein Block (Dako) before staining with primary antibodies; cTn T (2 μg/ml, rabbit polyclonal, Abcam) and CD31 (4 μg/ml, mouse monoclonal, clone JC70A, Dako) or vimentin (0.3 μg/ml, mouse monoclonal, clone V9, Dako), followed by Alexa Fluor-488-conjugated goat-anti-rabbit (10 μg/ml, Invitrogen) and Alexa Fluor-594-conjugated goat-anti-mouse (10 μg/ml, Invitrogen) secondary antibodies. Sections were then counterstained with 1 μg/ml of DAPI (Invitrogen) for nuclear staining. Epifluorescence images of immunostained sections were acquired with an Olympus BX61 upright microscope using analySIS software.

### Human iPSC NV Generation and Purification

Generation of NVs was performed as described (30, 31, 34) with modifications. Briefly, adherent iPSCs (CL2 and CERA) were individually harvested (following PBS wash) using a solution of EDTA (10 mM) (3 × 3 min round incubation) and cell suspension spun at 500 × *g* for 5 min. The pellet was resuspended in PBS and the cell suspension sequentially extruded through 10, 5, and 1 μm polycarbonate membranes (19 mm; Advanti Polar lipids, 610010) (13 times across each filter, Whatman). The extruded NVs were subsequently purified using 10% OptiPrep (Stemcell Technologies) density cushion (step gradient formed by overlaying extruded sample on 10% and 50% iodixanol) and centrifuged at 100,000 × *g* for 2 h at 4 °C. Seven equal fractions were obtained, diluted in PBS (to 1.5 ml), centrifuged at 100,000 × *g* for 2 h at 4 °C (TLA-55 rotor; Optima MAX-MP ultracentrifuge) and resuspended in PBS and stored at −80 °C until further use. The yield (protein) and density of each fraction was determined as described (44).

For comparison, natural EVs from each iPSC line were obtained as described previously (44), based on isopycnic (iodixanol density-based) ultracentrifugation (45, 46).

### NV Uptake in Engineered Cardiac Microtissues

To assess NV update in cardiac MTs, isolated NVs (30 μg/ml) were incubated with fluorescent dye DiI (Vybrant DiI Cell-Labeling Solution, 1:200 dilution, Invitrogen, V22885) at 1 μM concentration for 15 min at 37 °C as described (47). Briefly, dye-labeled NV and DiI control PBS (DiI) were centrifuged at 20,000 × *g* for 50 min on a 10% OptiPrep cushion, washed with PBS and resuspended in 50 μl of PBS. Labeled NVs and dye control were supplemented (v/v) on engineered cardiac MTs for 24 h following hypoxic exposure, fixed in 10% neutral buffered formalin for 1 h at RT and then dehydrated in 20% sucrose solution for 24 h. Dehydrated samples were embedded in Optimal Cutting Temperature compound (Sakura Finetek) and cryosections (10 μm thick) were treated with 0.1% Triton X-100 permeabilization buffer and Protein Block (Dako, Victoria, Australia) before staining with primary antibodies; cTn T (2 μg/ml, rabbit polyclonal, Abcam) and CD31 (4 μg/ml, mouse monoclonal, clone JC70A, Dako) or vimentin (0.3 μg/ml, mouse monoclonal, clone V9, Dako), followed by Alexa Fluor-488-conjugated goat-anti-rabbit (10 μg/ml, Invitrogen) secondary antibody. Sections were counterstained with 1 μg/ml of DAPI (Invitrogen) for nuclear staining. Epifluorescence images of immunostained sections were acquired with an Olympus BX61 upright microscope using analySIS software.

### *In vitro* Model of Hypoxia/Reoxygenation

Normoxia group was washed with PBS (3 x) and maintained in 0.4 ml of MT media at 37 °C in a humidified CO2 incubator (∼21% O_2_). Treatment groups (IRI UT, CL2 NVs T, and CERA NVs T) were washed with PBS (2×), and one wash with ischemic buffer ((in mmol/L: 1.0 KH2PO4, 10.0 NaHCO3, 1.2 MgCl2.6H20, and 25.0 Na(4-(2-hydroxyethyl)-1-piperazineethanesulfonic acid) (Hepes), 74.0 NaCl, 16.0 KCl, 1.2 CaCl2, and 10 mM 2-deoxyglucose, pH 6.7, gassed with pure nitrogen gas for 10 min). MTs were resuspended in 0.5 ml of ischemic buffer and incubated in a hypoxia incubator chamber (STEMCELL Technologies, 27310) and purged with nitrogen gas for 10 min (99% N_2_, 1% O_2_). The hypoxia incubator chamber was then placed inside an incubator at 37 °C for 2 h. Following hypoxia incubation, the ischemic media was removed, and 0.4 ml of complete MT media was added with either NVs treatment (CL2 or CERA) or PBS. All groups were incubated at normal oxygen conditions (5% CO_2_, O_2_ uncontrolled) for 24 h.

### Human cTnI ELISA

Quantification of cTnI in culture supernatant from normoxia and hypoxia/reoxygenation MTs was performed using an ELISA (Human cTn 1 SimpleStep ELISA Kit, Abcam, ab200016) according to manufacturer’s instructions. Briefly, MT culture media (quadruplicates per condition) was centrifuged at 2000*g* for 10 min to remove cell debris. A total of 50 μl of all sample (and standards) were added to the appropriate wells in duplicate. To each well, 50 μl of the Antibody cocktail was added and incubated for 1 h at RT on a plate shaker set to 400 rpm. All wells were washed three times with 1 x Wash Buffer PT. Next, 100 μl of TMB Development Solution was added to each well and incubated for 10 min in the dark on a plate shaker set to 400 rpm. The reaction was stopped by adding 100 μl of Stop Solution to all the wells, mixed on a shaker for 1 min. The optical density was then measured at 450 nm using a microplate spectrophotometer (Bio-Rad).

### Cardiac MT Contractility Assay

The contractility of MTs was recorded for normoxia and hypoxia, NV treated and NV untreated conditions, and analyzed using MUSCLEMOTION macro on ImageJ, as described before (48). Briefly, capture videos of all MTs (n = 6) were taken using a high FPS camera (Olympus DP72, set to 15 frames per second) adapted to an inverted microscope (Olympus IX71), at 200× magnification. All videos were analyzed using the MUSCLEMOTION macro at a speed window of 2 frames (15 FPS). For those MTs which contraction propelled them out of the frame of evaluation, manual generation of stacks was required (as instructed on the original paper). Data were plotted on Graph Pad Prism V.10.4.2.

### Proteomics: Solid-phase-enhanced Sample Preparation

Normoxia MTs proteome n = 4, hypoxia MTs proteome n = 4, CL2 NV hypoxia proteome n = 4 and CERA NV hypoxia MT proteome n = 4 were lysed in 1% v/v SDS, 50 mM triethylammonium bicarbonate, pH 8.0, incubated at 95 °C for 5 min and quantified by microBCA (Thermo Fisher Scientific, 23235) as described (49). Proteomic sample preparation was performed from 10 μg of protein extract as previously described (47) using single-pot solid-phase-enhanced sample preparation (SP3) (50). Briefly, samples were reduced with 10 mM DTT at RT for 1 h (350 rpm), alkylated with 20 mM iodoacetamide (IAA) (Sigma-Aldrich) for 20 min at RT (light protected), and immediately quenched with 10 mM DTT. A Sera-Mag SpeedBead carboxylate-modified magnetic particle mixture (hydrophobic and hydrophobic 1:1 mix, 65152105050250, 45152105050250, Cytiva) were added to each protein extract, washed in 50% ethanol, and incubated for 10 min (1000 rpm) at RT. Beads were sedimented on a magnetic rack, supernatants removed and beads washed three times with 200 μl 80% ethanol. Beads were resuspended in 100 μl 50 mM triethylammonium bicarbonate pH 8.0 and digested overnight with trypsin (1:50 trypsin: protein ratio; Promega, V5111) at 37 °C, 1000 rpm. The peptide and bead mixture was centrifuged at 20,000*g* for 1 min at RT. Samples were placed on a magnetic rack and supernatant was collected and acidified to a final concentration of 1.5% formic acid, frozen at −80 °C for 20 min, and dried by vacuum centrifugation for ∼1 h. Peptides were resuspended in 0.07% TFA, quantified by Fluorometric Peptide Assay (Thermo Fisher Scientific, 23290) as per manufacturer’s instructions, and samples normalized with 0.07% TFA.

### Proteomics: Nano Liquid Chromatography–Tandem Mass Spectrometry

Peptides were analyzed on a Dionex UltiMate NCS-3500RS nanoUHPLC coupled to a Q-Exactive HF-X hybrid quadrupole-Orbitrap mass spectrometer equipped with nanospray ion source in positive, DDA mode (51, 52). Peptides were separated using high resolution analytical nano liquid chromatography separated (1.9-μm particle size C18, 0.075 × 250 mm, Nikkyo Technos Co. Ltd) with a gradient of 2 to 28% acetonitrile containing 0.1% formic acid over 95 min at 300 nl min-1 followed by 28 to 80% from 95 to 98 min at 300 nl min-1 at 55 °C (butterfly portfolio heater, Phoenix S&T). An MS1 scan was acquired from 350–1650 m/z (60,000 resolution, 3 × 10^6^ automatic gain control, 128 msec injection time) followed by MS/MS DDA (top 25) with collision-induced dissociation and detection in the ion trap (30,000 resolution, 1 × 10^5^ automatic gain control, 60 ms injection time, 28% normalized collision energy, 1.3 m/z quadrupole isolation width). Unassigned, 1, 6 to 8 precursor ions charge states were rejected and peptide match disabled. Selected sequenced ions were dynamically excluded for 30 s. Data were acquired using Xcalibur software v4.0 (Thermo Fisher Scientific). The MS-based proteomics data and analysis parameters have been deposited into the ProteomeXchange Consortium *via* the MassIVE partner repository with the dataset identifier MSV000101964.

### Proteomics: Data Processing and Informatics/Visualization

Identification and quantification of peptides was performed using MaxQuant (v1.6.14.0) with its built-in search engine Andromeda (53), as described (49, 51, 54). Human-only (UniProt # 78,120 entries) sequence database (03/22) with a contaminants database was employed. N-terminal acetylation and methionine oxidations were set as variable modifications. FDR was 0.01 for protein and peptide levels. Enzyme specificity was set as C-terminal to arginine and lysine using trypsin protease, and a maximum of two missed cleavages allowed. Peptides were identified with an initial precursor mass deviation of up to 7 ppm and a fragment mass deviation of 20 ppm. Protein identification required at least one unique or razor peptide per protein group. Contaminants, and reverse identification were excluded from further data analysis. “Match between run algorithm” in MaxQuant (55) and label-free protein quantitation (maxLFQ) was performed. All proteins and peptides matching to the reversed database were filtered out.

Perseus (56) and R studio were used to analyze the proteomic data and generate plots. G:Profiler, Reactome, STRING were used for enrichment analysis. Protein lists for samples were generated in Perseus (v1.6.14.0) (57). For hCMT proteomes, proteins were identified at least three out of four replicates (>70%) per group. Protein intensities (maxLFQ) were log2 transformed. Statistical analysis and plots were generated using Perseus, R studio and GraphPad Prism. PCA, Pearson correlation matrix, and hierarchical clustering was performed in R studio using Euclidian distance and average linkage clustering, with missing values imputed at z-score 0 for heatmap generation only. R was used for data visualization (ggplot2, ggpubr packages). In all instances significance was *p* < 0.05 unless otherwise indicated.

### Proteomic GSEA and Cardiac Cell Type Reference Mapping

To resolve cell type-specific proteomic alterations and determine microenvironmental patterns driving cardiac remodeling, proteomic differential expression profiles were projected onto a myocardial IRI single-cell transcriptomic reference framework (58). Canonical cluster marker genes defined by Molenaar *et al*. *via* localized differential gene expression analysis across sequential postinjury phases were compiled into reference gene sets. These sets captured active, phase-specific transcriptional profiles spanning baseline, acute ischemic, and active remodeling states.

To evaluate cell-type representation, GSEA was performed for differential proteome analyses of each NV group relative to IR (clusterProfiler in R). Here, enrichment algorithm calculated a running sum Kolmogorov-Smirnov statistic to derive NES for each scRNA-seq cell cluster, mapping specific cell-type markers that align with ranked proteomic distribution, and statistical significance adjusted for multiple testing using the BH FDR method, with a significance threshold at p.adj <0.05. Global distribution profiles were visualized *via* multi-variable dot plots, with probability density distributions of enrichment scores provided across the distinct cellular niches modeled *via* ridge plots to isolate treatment-specific cellular assignment. For specific proteins that underlie the cell-type shifts identified, individual proteins contributing to the leading-edge core enrichment signal were isolated, mapped, and annotated to their specific coordinates on the comprehensive rank-ordered fold-change curves.

## Results

### Modeling Ischemia-Reperfusion Injury with Human iPSC-Derived Cardiac MTs

Cardiac cell differentiation was performed using the human iPS-Foreskin-2 (CL2) cell line (39), generating CMs, endothelial cells, and cardiac fibroblasts (*via* the epicardial lineage), as detailed in Experimental Procedures. We have shown that these cardiac MTs display multicellular organization and beating frequencies characteristic of the developing human heart, with vascularization and interspersed interstitial fibroblasts/mesenchymal-like cells among the CMs (12). To construct the cardiac MTs, enriched day-19 iPSC-derived CMs (1.2 × 10^5^ cells/well) were seeded onto Matrigel-coated UpCell plates, and iPSC-derived endothelial cells (7 × 10^4^ cells/well) and iPSC-derived cardiac fibroblasts (1 × 10^4^ cells/well) then seeded 24 h following.

**Fig. 1:**
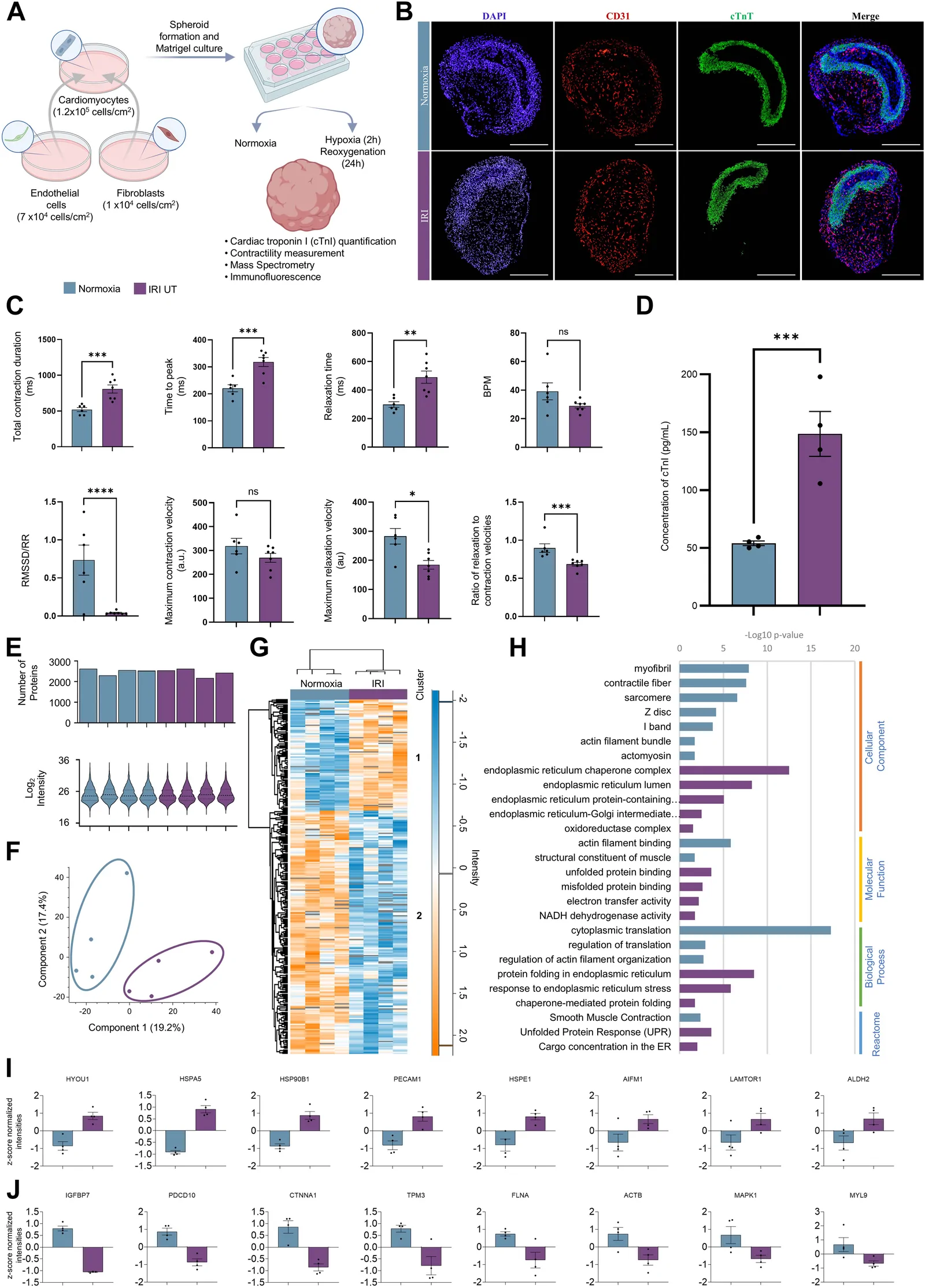
**Human cardiac microtissue simulated IRI model.***A*, schematic overview of cardiac microtissue generation, culture, and hypoxia-reoxygenation workflow, with associated functional and analytical readouts. Human cardiac microtissue were generated from defined populations of human induced pluripotent stem cell-derived cardiomyocytes (60%, 120,000 cells), endothelial cells (35%, 70,000 cells), and cardiac fibroblasts (5%, 10,000 cells). Cells were assembled as a detached cell sheet, transferred to suspension for spheroid formation, and embedded in Matrigel. IRI was modeled by simulated ischemia (2 h, 1% oxygen, N_2_-gassed ischemic buffer) followed by reperfusion under normoxic conditions in complete microtissue media for 24 h. Cardiac microtissues were assessed for cell viability, contractile function, and molecular changes using immunofluorescence and mass spectrometry-based proteomics. *B*, representative immunofluorescence imaging of human cardiac microtissues: Normoxia and hypoxia/reoxygenation conditions. The scale bar represents 500 nm. DAPI, CD31, cardiac troponin T (cTnT). *C*, quantification of contractility parameters (normoxia n = 6, IRI n = 7). Error bars indicate SEM. *D*, Cardiac troponin I (cTnI) release measured by ELISA (n = 4). Error bars indicate SEM. *E*, microtissue proteomic coverage and protein abundance (LFQ normalized intensity) for each group (n = 4). *F*, principal component analysis of cardiac microtissue proteome. *G*, hierarchical cluster analysis of normoxia and IRI microtissue samples identifies distinct protein expression profiles (ANOVA, *p* < 0.05, fold change ± 2.0). *H*, Gene Ontology enrichment (cellular component, molecular function, and biological process), and Reactome pathway enrichment analyses of differentially expressed proteins. *I* and *J*, representative proteins significantly upregulated (*I*) or downregulated (*J*) in response to IRI relative to normoxic/vehicle control based on -log10 *p* value. Error bars indicate SEM. DAPI, 4′,6-diamidino-2-phenylindole; IRI, ischemia-reperfusion injury; LFQ, label-free quantification.

These cardiac MT sheets were cultured (24 h), transferred, embedded, and cardiac MTs then embedded in growth factor reduced Matrigel and cultured in cardiac MT medium, designated as day 0 (media changed every 2–3 days), before 7 to 10 days prior to ischemic conditioning and any functional assessments performed. An *in vitro* model of hypoxia/reoxygenation was developed, involving a sequential hypoxia incubation with ischemic media, followed by reoxygenation with complete culture media, to simulate tissue injury caused by sudden reoxygenation (reperfusion injury). Normoxic hCMT (normoxia), and IRI cardiac MT, (hypoxia-reoxygenation) conditions were then established and immunofluorescence confocal microscopy, cTnI quantification, and functional assessment analyses performed on this model (Fig. 1*A*).

To visualize cell lineage markers associated in cardiac MTs, immunofluorescence confocal microscopy revealed vimentin (fibroblast), CD31 (endothelial) and cTn (CM) expression, which are the major cell types present in the myocardium *in vivo* (Fig. 1*B*). Contractile function assessment was then performed to assess functional impacts following IRI assay on cardiac MT. Our data highlight significant differences in total contraction duration (*p* < 0.001), time to peak (*p* < 0.0005), relaxation time (*p* < 0.005), maximum relaxation velocity (*p* < 0.05), ratio of relaxation to contraction velocities (*p* = 0.0005), and the Root Mean Square of Successive Differences (RMSSD) normalized to the R-R interval (*i.e*., RMSSD/RR) (*p* < 0.0001), commonly used in heart rate variability (HRV) analysis (Fig. 1*C*). The latter being the clinical parameter which provides insights into the balance between the sympathetic and parasympathetic nervous systems, which can be affected by various factors like stress, exercise, and sleep (59). Changes in HRV, including RMSSD, have been linked to cardiovascular health and other conditions (59, 60). To further evaluate the extent of tissue damage, cTnT release was performed, used clinically to detect suspected myocardial damage. hCMT subjected to IRI resulted in 3-fold increase in cTnT (*p* < 0.0005) in the conditioned media compared with normoxia controls (Fig. 1*D*). These findings confirm that IRI of hCMT induces functional perturbation and CM death.

### Cardiac MT Proteome Remodeling with Ischemia/Reperfusion Injury

To understand the cellular changes in human cardiac MT in response to hypoxia/reoxygenation, we performed proteomic profiling. Proteomic analysis was performed on cardiac MT in N = 4 during the culture period. Samples were lysed in SDS and reduced/alkylated before being subjected to protein hydrophobic and hydrophilic capture-based tryptic digestion (38) and nLC-MS analysis. Proteins were subjected to direct DDA MS combined with label-free quantitation (31, 37), processed through MaxQuant and analyzed for reproducibility and quality assessment. In total, more than 2600 proteins were identified (Supplementary Tables S1–S3). Of these identified proteins, 2396 proteins could be quantified across normoxia and hypoxic conditions; 2506 normoxia and 2505 in hypoxic condition (identified in at least three out of four replicates (>70%) per group) (Fig. 1*E*, Supplementary Tables S1–S3). PCA revealed the separation of cardiac MT proteomes in response to IRI modeling (Fig. 1*F*, Supplementary Tables S1–S3). To confer cardiac identity, we report 6/12 highly enriched heart tissue expressed proteins based on Human Protein Atlas (61) including actin and myosin binding proteins MYL4, MYH6, MYL7, MYBPC3, and heart muscle troponins TNNI3, TNNT2. We further report coverage of heart cell markers in this cardiac MT proteome, including cardiac fibroblasts (6 proteins; *e.g*., COL12A1, COL6A2, MRC2, and FBLN1), CMs (29 proteins; *e.g*., FABP3, MYL3, SDHA, TNNT2, and MYBPC3), cardiac endothelial cells (1 protein; CD93), and cardiac smooth muscle cell protein MYH11 (Supplementary Tables S1 and S2).

We performed differential analysis of this cardiac MT proteome in response to IRI and detected 293 proteins that were significantly dysregulated in expression (normalized protein expression data, hierarchical K-means clustering, FDR <0.05, Fig. 1*G*, Supplementary Table S4; 92 upregulated, and 201 downregulated proteins. Importantly, we validate key cardiac cell markers in response to IRI indicating a remodeling and expression change induced by hypoxia in this assay, such as TPM1, MYL9, FLNA, and MYOM1, CMs markers (components of z-disc and contractile fiber), decreased in expression indicating a relative loss in CM composition/function. Gene Ontology-/Reactome-term annotation of the respective proteins and clusters revealed enrichment in normoxia proteome associated with cardiac structure (myofibril, contractile fiber, sarcomere, Z disc, I band, actin filament bundle, and actomyosin), cardiac molecular function (structural constituent of muscle and actin filament binding), cardiac tissue regulation (regulation of actin filament organization), and smooth muscle contraction (Fig. 1*H*, blue bars, Supplementary Table S9). In response to IRI, cardiac MT proteome cluster reflected enrichment in endoplasmic reticulum (ER) stress (ER chaperone complex, ER protein-containing complex, ER-Golgi intermediate compartment), oxidoreductase complex, networks associated with unfolded/misfolded proteins, electron/NADH transfer activity, and unfolded protein response (Fig. 1*H*, purple bars, Supplementary Table S9). The data confirmed the patterns of an increase in intracellular stress networks, altered matrix protein networks and a decrease in cardiac cell composition linked with contractile functional protein networks in the heart with IRI.

Within these distinct cardiac MT proteome clusters in response to IRI, we identified several key proteins upregulated in expression associated with hypoxia response (HYOU1) (62), heat shock proteins (HSPA5/90B1/E1) (63, 64), metabolic and energetic change (ALDH2, NDUFB3, HAGH, and UQCRC2) (65, 66, 67, 68), and apoptosis (AIFM1) (69), previously attributed in response to myocardial IRI (34, 60, 70, 71) (Fig. 1*I*, Supplementary Table S4). In contrast, select proteins downregulated in response to IRI were involved in cell/tissue/matrix homeostasis, structural organization, and contractile function (TPM1, CTNNA1FABP3, COL15A1, ACTB, VIM, COL18A1, MYL7/9/12A, MYOZ2, and COLGALT1) (34, 60, 70, 71) (Fig. 1*J*, Supplementary Table S4). These findings demonstrate the patterns of an increase in hypoxia and stress response proteins, apoptotic regulators, and changes in metabolic and energetic protein networks, while a decrease in extracellular matrix (ECM) organization, contractile response, and cytokine signaling proteins.

### Functional NVs Protect Human Cardiac MTs Against Simulated Ischemia-Reperfusion Injury

**Fig. 2:**
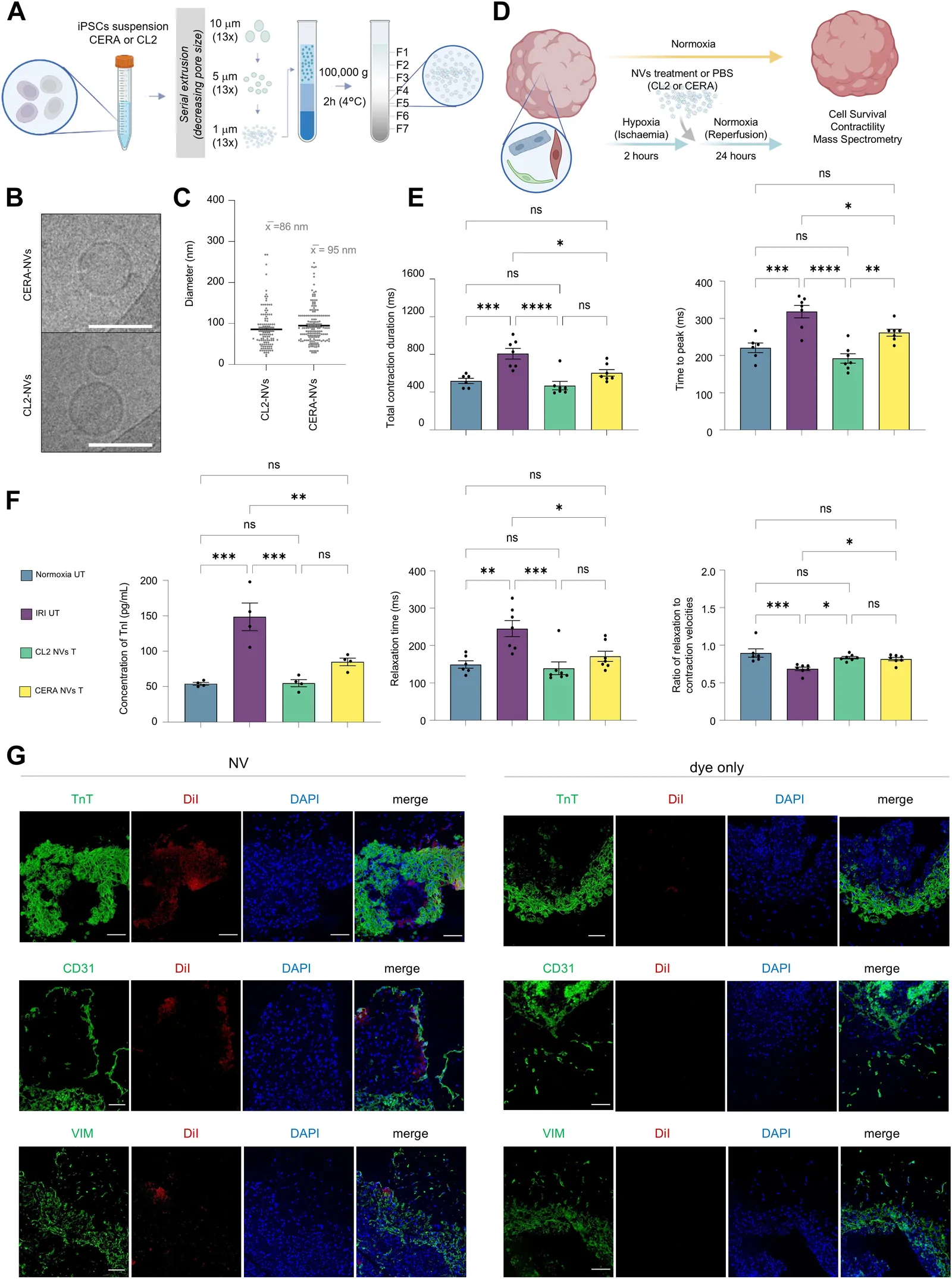
**Generation of functional nanovesicles (NVs) from human iPSCs to protect against IRI-induced cell death and preserve contractility in human cardiac microtissues.***A*, human iPSC-derived NVs were generated by serial extrusion through 10, 5, and 1 μm polycarbonate membranes (13 extrusion cycles per membrane) and purified by density-cushion ultracentrifugation. The NV-enriched fraction (F5) was used for all subsequent biophysical characterization and functional analyses. NVs were derived from two different hiPSC lines (CL2 and CERA). *B*, representative cryo-electron microscopy images showing spherical NV morphology. The scale bar represents 100 nm. *C*, size distribution for NVs derived from CL2 and CERA iPSCs. *D*, experimental workflow for single-dose NV (or PBS) treatment of cardiac microtissues in the hypoxia-reoxygenation assay. Following 24 h of reoxygenation, microtissues were analyzed for cell viability, contractility, and proteomic remodeling. *E*, quantification of contractility parameters in NV-treated groups (CL2 NVs T and CERA NVs T) and NV-untreated groups (Normoxia UT and IRI UT). Normoxia UT n = 6, all other groups n = 7. *F*, cardiac troponin I (cTnI) levels measured by ELISA (n = 4). Data presented as mean ± SEM. ∗*p* < 0.05, ∗∗*p* < 0.005, ∗∗∗*p* < 0.001, ∗∗∗∗*p* < 0.0001, ns = nonsignificant. *G*, internalization of NVs (CERA) by cardiac microtissue simulated IRI model. NV uptake and interaction with specific cell types using troponin/TnT (cardiomyocyte), CD31 (endothelial), and vimentin/VIM (fibroblast) relative to DiI only control. Fluorescence microscopy analysis of hCMT model incubated with NVs labeled with lipophilic dye (DiI) for 24 h. DiI vehicle (PBS) control. Nuclei (*blue*) were stained with DAPI. The scale bar represents 100 μm. iPSC, induced pluripotent stem cell; IRI, ischemia-reperfusion injury.

To assess the therapeutic regulation of stem cell-derived NVs in cardiac MT in response to IRI, we generated NVs from different iPSC lines and applied functional and proteome analyses to understand this system as a functional regulation platform. We have previously demonstrated that NVs, generated directly from human iPSCs, influence cardiac tissue and functional repair mechanisms in 2D cardiac cellular models (34). NVs were generated from two different human iPSC lines (CERA and CL2), their membrane components was purified and biophysical and biochemical characterization was performed based on their size, morphology and protein markers as previously described (34) (Fig. 2*A*). Cryogenic transmission electron microscopy analysis revealed that particle size and distribution were similar between 2 cell sources (range 30–250 nm), with mean diameter of 86 nm (CERA) and 95 nm (CL2) (Fig. 2, *B* and *C*).

Building on insights of proteome organization of our cardiac MT model in response to IRI, we aimed to define NV-mediated response in this model. Here, treatment groups included normoxia NV-untreated (normoxia UT and PBS/vehicle), and IRI groups: Hypoxia-reoxygenation untreated (hypoxic vehicle control: IRI UT), and NV treatment groups (CL2 NVs T and CERA NVs T). IRI groups were incubated for 2 h in hypoxic media (N_2_ pregassed), followed by 24 h in reoxygenation media supplemented with either NVs (single dose, 30 μg/ml, based on prior reported functional response in cardiac cells (34)) or vehicle control (PBS) (Fig. 2*D*).

To assess functional impact of NVs on hCMT model of IRI, contractility analyses was performed as previously described (12, 16). Here, we confirmed that our IRI model significantly impairs the functional capacity of hCMT compared to normoxia condition, including lengthening of total contraction duration (*p* < 0.001), extended time-to-peak (*p* < 0.0005), and increased relaxation time (*p* < 0.005). NV treatments mitigated these contractile perturbations induced by IRI (Fig. 2*E*). Importantly, NV-treated hCMT exhibited relaxation-contraction velocity ratios comparable to normoxia controls, indicating improved diastolic (relaxation) function (72) (Fig. 2*E*). To evaluate whether NVs also provide prosurvival benefits, cTnT concentrations in conditioned media were measured. ELISA analysis showed that NVs from both CL2 and CERA iPSC lines significantly reduced cell death compared to vehicle-treated IRI group (CL2 NVs, *p* < 0.005, CERA NVs, *p* < 0.0005) (Fig. 2*F*). These results confirm that distinct iPSC-derived NVs modulate the functional capacity and survival responses of hMCTs under hypoxic and reoxygenation conditions.

Internalization of NVs by cardiac MT simulated IRI model was established. Here, NVs were labeled with lipophilic tracer DiI and isolated using cushion-based centrifugation to separate unbound dye (44). Fluorescence microscopy revealed NV uptake and interaction with specific cell types using troponin/TnT (CM), CD31 (endothelial), and vimentin/VIM (fibroblast) relative to DiI only control after 24 h (Fig. 2*G*).

### NV-mediated Remodeling of Cardiac MT Proteome in Ischemia-Reperfusion Injury Model

**Fig. 3:**
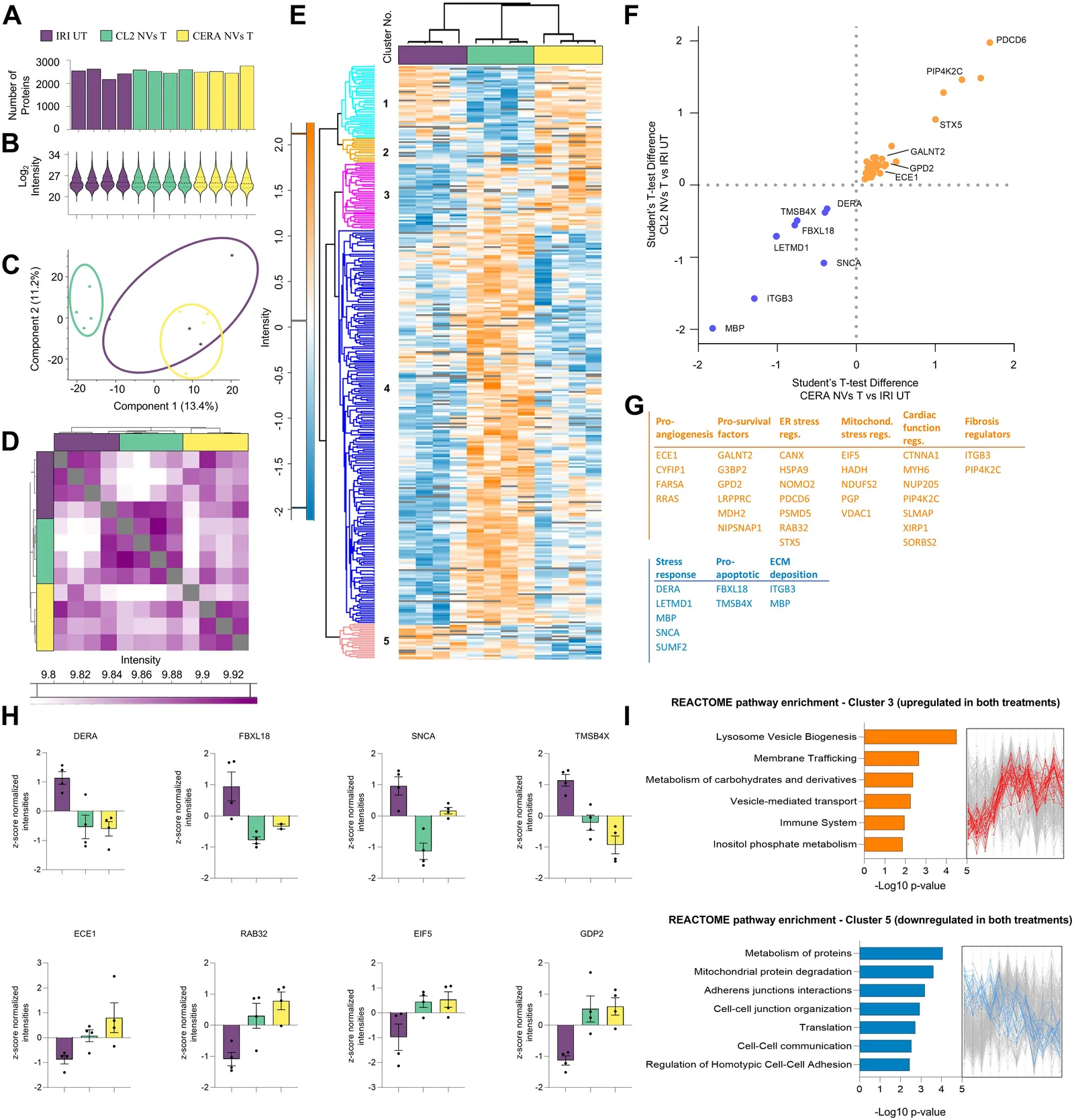
**NVs were conserved in their proteome remodeling of cardiac microtissue model of simulated IRI model.***A* and *B*, for organoid and NV-treated organoid groups, protein coverage/identification and abundance (LFQ normalized intensity) and (*C*) principal component analysis is shown (n = 4). *D*, Pearson Correlation Analysis (R Score) of IRI UT, CL2 NVs T, and CERA NVs T (n = 4). (*E*) Hierarchical cluster analysis of IRI UT, CL2 NVs T and CERA NVs T (*p* < 0.05, fold change ± 2.0), reveals 5 clusters of proteins differentially expressed. *F*, scatter plot for unique pro-reparative factors conserved in expression differences for both NV-treated groups. Student *t* test difference, *p* < 0.05. *G*, differentially expressed proteins either up or downregulated following NV treatment that are associated with pro-reparative/wound healing function. *H*, differential protein abundance (fold change, of LFQ intensity, log2) of selected protein markers for each group, relative to IRI UT (control) (*purple*) (*p* < 0.05). Select proteins include prosurvival, proangiogenic, endoplasmic reticulum and mitochondrial stress regulators, and stress protein responders identified. *I*, enrichment analysis (ranked, *p* < 0.05) for Reactome pathway enrichment and protein intensity profile rank for Clusters 3 (upregulated in both treatments) and 5 (downregulated in both treatments). IRI, ischemia-reperfusion injury; LFQ, label-free quantification; NV, nanovesicle.

Cardiac MT proteomic response to NV treatment was performed. Here, after 24 h following NV treatment in IRI assay (and vehicle hypoxic control), cardiac MT lysates were collected and proteome profiling performed. Proteome analyses revealed a network of proteins identified in each group and differential proteome clustering across groups confirmed by PCA and Pearson’s correlation analyses (Fig. 3, *A*–*D*, Supplementary Table S5). Differential analysis by K-means clustering (*adj p* < 0.05) revealed 5 distinct clusters of protein networks based on expression in cardiac MT proteome, and a conserved NV differential response protein signature 58 in response to IRI (Fig. 3*E*, Supplementary Table S5).

We initially focused on NV-mediated remodeling conserved between both iPSC sources (i.e., CL2 and CERA NVs). Proteome analyses of cardiac MT proteome (NVs *versus* IRI) revealed statistically significant (*adj p* < 0.05) upregulation of proteins involved in angiogenesis (ECE1 and FARSA) (73, 74), prosurvival (G3BP2, GPD2, RPPRC, MDH2, and NIPSNAP1) (75, 76, 77, 78, 79), ER stress regulation (HSPA9, NOMO2, PSMD5, RAB32, and STX5) (80, 81, 82, 83, 84), mitochondrial stress regulation (EIF5, HADH, NDUFS2, PGP, and VDAC1) (85, 86, 87, 88), cardiac function (CTNNA1, MYH6, NUP205, PIP4K2C, SLMAP, SIRP1, and SORBS2) (89, 90, 91, 92, 93, 94, 95), and regulators of fibrosis (ITGB3) (96) (Fig. 3, *F*–*H*, Supplementary Tables S8 and S14).

We observed further downregulated protein networks in response to NV treatments including key factors related to stress response (DERA, LETMD1, MBP, SNCA, and SUMF2) (97, 98, 99, 100, 101), apoptosis promoters (FBXL18 and TMSB4X) (102, 103) and ECM deposition (MBP) (104). GO-/Reactome-based enrichment analysis for cluster 3 (upregulated in both NV treatment groups relative to IRI vehicle) highlighted enrichment in networks associated with lysosome vesicle biogenesis, membrane trafficking, metabolism of carbohydrates and derivatives, vesicle-mediated transport, immune system, and inositol phosphate metabolism (Fig. 3*I*, top panel, Supplementary Table S10). Downregulated protein cluster (cluster 5) showed enrichment in networks of cellular stress such as protein metabolism, mitochondrial protein degradation, regulation in adherens junctions, cell-cell junction organization, and cellular communication (Fig. 3*I*, bottom panel, Supplementary Table S10). Importantly, we showed that NVs mediate specific changes in cardiac marker expression in response to IRI, including upregulated expression of various cardiac-associated signaling and structural networks proteins, including phosphatase PGP, ion channel VDAC1, translation factor DRG2, and dehydrogenase HADH. These results align with the functional data obtained further supporting the reprogramming capacity of functional NVs in cardiac MT.

### Selective NV Reprogramming of Cardiac MT Proteome Based on Cell Source

**Fig. 4:**
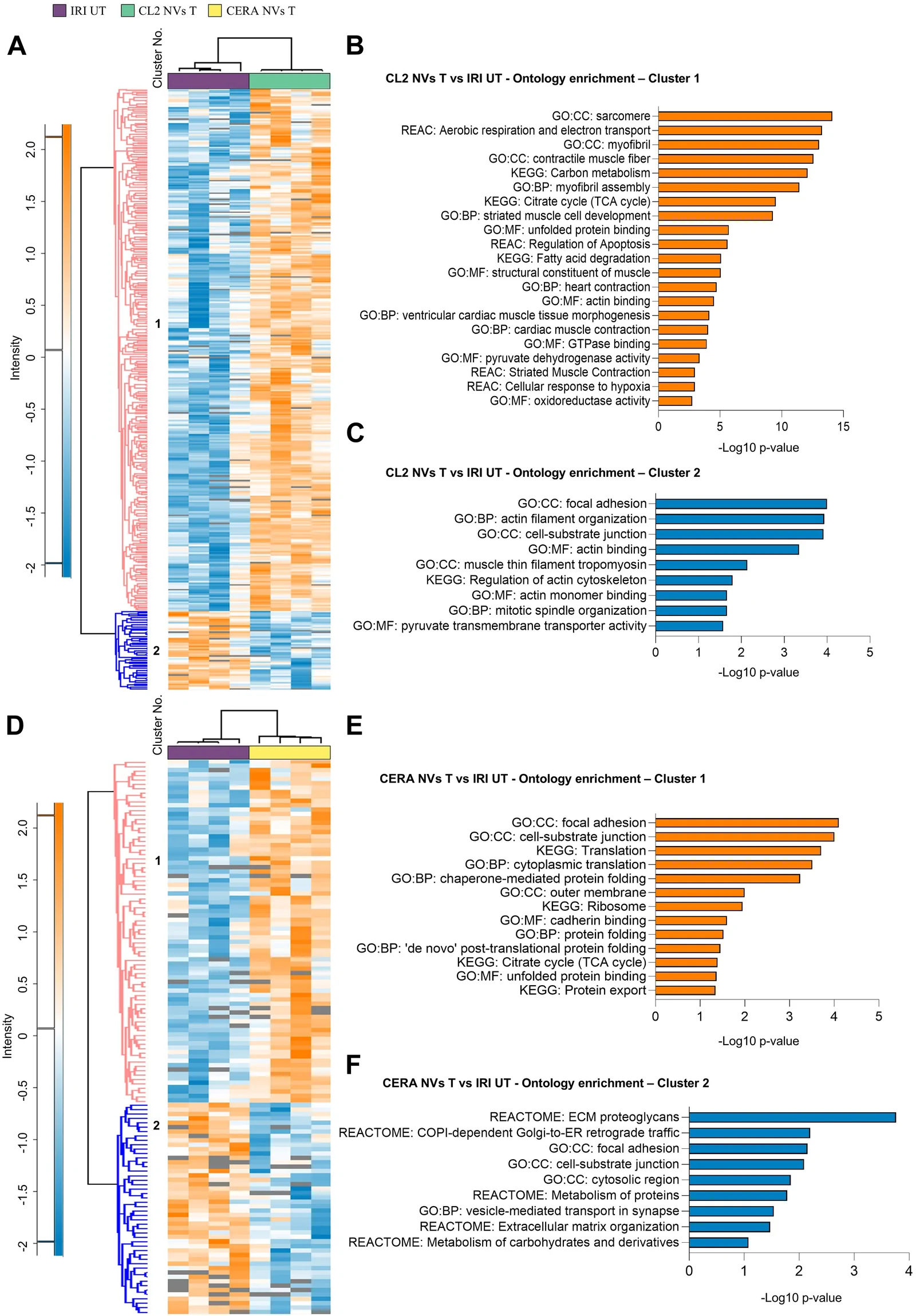
**Human iPSC origin confers selective NV-mediated remodeling of cardiac microtissues.***A*, hierarchical cluster analysis of CL2 NVs T and IRI UT (*p* < 0.05, fold change ± 2.0), reveals distinct clusters of differential protein expression. *B*, functional enrichment analysis (ranked, *p* < 0.05) of CL2 NVs T and IRI UT for Reactome pathway enrichment, Cellular Component, KEGG pathways, Molecular Function, Biological Process for Cluster 1. *C*, enrichment analysis (ranked, *p* < 0.05) of CL2 NVs T and IRI UT for Cluster 2. *D*, hierarchical cluster analysis of CERA NVs T and IRI UT (*p* < 0.05, fold change ± 2.0). *E*, functional enrichment analysis s (ranked, *p* < 0.05) of CERA NVs T and IRI UT for Cluster 1. *F*, enrichment analysis (ranked, <0.05) of CERA NVs T and IRI UT for Cluster 2. iPSC, induced pluripotent stem cell; NV, nanovesicle; IRI, ischemia-reperfusion injury; KEGG, Kyoto Encyclopedia of Genes and Genomes.

We next sought to elucidate differences in NV potency (Fig. 2, *E* and *F*) by examining proteome reprogramming induced by each NV source in the hCMT assay. Here, K-means clustering analysis revealed distinct cluster subsets for CL2-NV (Fig. 4*A*) and CERA-NV (Fig. 4*D*) relative to IRI vehicle control (Supplementary Tables S6 and S7). Enrichment analysis revealed NVs from CL2 iPSCs (cluster 1) (Fig. 4*B*, Supplementary Table S11) involved in myofibril assembly, striated muscle cell development, cardiac (chamber/ventricle) muscle tissue morphogenesis, cardiac muscle contraction, and heart morphogenesis. Further enriched functions were associated with actin binding, structural constituent of muscle, unfolded protein binding and energy production (GTPase and ADP binding, pyruvate dehydrogenase activity, oxidoreductase activity). Reactome analysis showed aerobic respiration and respiratory electron transport, TCA cycle, regulation of apoptosis. Finally, we observed an enrichment in KEGG pathways involved in carbon metabolism, Citrate cycle (TCA cycle), and fatty acid degradation. Ontology analysis of cluster 2 (Fig. 4*C*, Supplementary Table S11) showed a downregulation of cellular components involved in focal adhesion and muscle thin filament tropomyosin, biological processes linked to actin filament organization and mitotic spindle organization, and molecular function in actin binding and pyruvate transmembrane transporter activity, with pathways analysis decreased in regulation of actin cytoskeleton.

CERA iPSC-derived NVs (cluster 1) (Fig. 4*E*, Supplementary Table S12) revealed enrichment of pathways related to cytoplasmic translation and protein folding, including cadherin binding and unfolded protein binding. Intracellular networks were enriched for mitochondrial outer membrane, focal adhesion, and cell-substrate junction components, with functional associations linked to voltage-gated anion channel activity, ceramide binding, and metabolic response. In contrast, proteins downregulated in response to CERA-NVs treatment relative to the IRI vehicle control (cluster 2) (Fig. 4*F*, Supplementary Tables S12 and S13) were enriched for focal adhesions, cell-substrate junctions, ECM organization and proteoglycans, as well as metabolism of carbohydrates and derivatives.

These differences in NV-mediated proteomic remodeling of hCMT indicate that, while NVs from different iPSC lines broadly enhance functional capacity and promote a pro-reparative cardiac response, the extent and nature of this remodeling are cell-source dependent. Specifically, CL2-NVs reprogram hCMT in the IRI assay towards sustained basal cardiac function processes, corresponding with greater functional potency, whereas CERA-NVs predominantly modulate cellular stress responses and mitochondrial function networks.

### Cell Type-specific Proteomic Remodeling of hCMT by Different NVs in Ischemic Myocardium

**Fig. 5:**
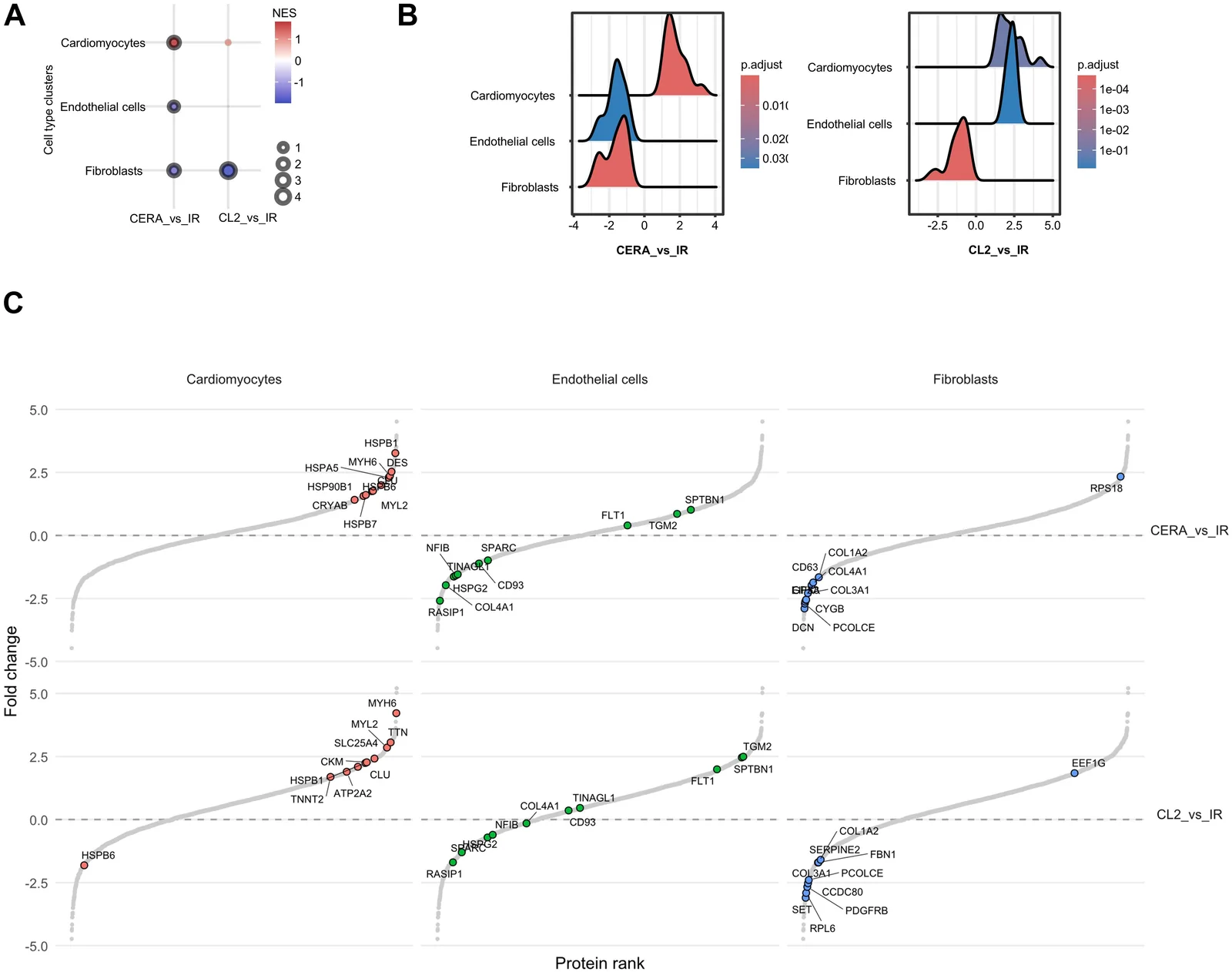
**NV-mediated remodeling of cardiac microtissues reveals changes in the cellular composition in response to hypoxic injury.***A*, proteome distribution of hCMT in response to NV treatment from different iPSC source. Dot plot representing GSEA-based cell type enrichment for cardiomyocytes, endothelial cells, and fibroblasts under CERA *versus* IR and CL2 *versus* IR conditions. Color scale represents Normalized Enrichment Score (NES), where size of each circle correlates with statistical significance (-\log10 *adj p*). *B*, ridge plots indicating distribution of enrichment scores and probability densities for specific cardiac cell type clusters in response to NV treatment; CERA NVs (*left*) and CL2 NVs (*right*). *C*, gene/protein-rank distribution curves for specific cardiac cell types, where key differentially expressed regulatory proteins are highlighted. Reference single-cell transcriptomic marker profiles and cell-type designations were defined using the mouse myocardial ischemia/reperfusion injury (IRI) single-cell reference dataset (58). GSEA, gene set enrichment analysis; hCMT, human cardiac microtissue; iPSC, induced pluripotent stem cell; NV, nanovesicle.

To resolve the cell type-specific impacts of NVs from CERA and CL2 source against IRI in hCMT assay, we performed GSEA utilizing cell-type marker profiles from a reference myocardial IRI dataset resource (58) (Supplementary Table S15 and S16). Global enrichment profiles revealed distinct, divergent cell-type responses between NV treatments (Fig. 5, *A* and *B*). Under CERA *versus* IRI conditions, CMs exhibited a marked positive enrichment, suggesting a preservation or recovery of metabolic and structural CM processes. Conversely, both endothelial cells and fibroblasts displayed negative enrichment trends, indicating a suppression of injury-induced activation in these non-myocyte populations. Interestingly, CL2 *versus* IRI comparison demonstrated a contrasting signature, characterized by a profound downregulation/negative enrichment within the fibroblast cluster, alongside stable or minimally altered profiles in the parenchymal fractions. Furthermore, we highlight density shifts confirmed that while CERA NVs predominantly drives positive translational shifts within the CM compartment, CL2 NVs exerts a highly coordinated, repressive pressure on the mesenchymal and endothelial transcriptomic/proteomic machinery (Fig. 5*B*).

To pinpoint the key molecular effectors driving these cell-type-specific enrichment patterns, we mapped individual protein fold-changes across ranked distribution curves for each isolated cluster (Fig. 5*C*, Supplementary Table S16). Within the CM fraction, CERA NV treatment upregulated crucial structural and cytoprotective proteins, including *Myh6*, *Des*, *Clu*, and several small heat shock proteins (*Hspb1*, *Hspa5*, *Hspb6*, and *Hspb7*). In contrast, CL2 NVs *versus* IR CMs displayed a downregulation of *Hspb6*, despite maintaining elevations in structural transcripts like *Myl2* and *Ttn*. Within the endothelial cell compartment, both treatments effectively suppressed a core network of angiogenic and ECM remodeling factors—including *Flt1*, *Col4a1*, and *Cd93*—while selectively modulating transglutaminase 2 and spectrin. Finally, the fibroblast compartment revealed a stark, treatment-specific remodeling of the cardiac interstitium. Under CERA NVs, fibroblasts exhibited an accumulation of specific structural components (*e.g*., *Col1a2*, *Col4a1*) but a suppression of decorin and procollagen C-endopeptidase enhancer. Meanwhile, CL2 treatment triggered a comprehensive crash in fibrotic driving machinery within the fibroblast rank curve, heavily downregulating *Col1a2*, *Col3a1*, *Serpine2*, *Fbn1*, and *Pcolce*. Taken together, these data further support the cell type specific functional remodeling mediated by NVs.

## Discussion

Here, we describe a comprehensive analysis of proteins expressed by human iPSC-derived cardiac MT and tie these findings to proteome changes that occur relative to therapeutic intervention by NVs, fundamental knowledge needed to advance the use of cardiac MT to model human disease and molecular insights towards deliverable cell-free therapeutics. We demonstrate that NVs tune multiple pro-tissue repair networks, including cardiac structural and function, as well as regulators of cellular stress and metabolism (34, 60, 70, 71). While the therapeutic potential of these protein networks often relies on direct delivery to the heart to reactivate regenerative pathways in adult CMs, NVs provide a potent and deliverable strategy to translate such regulation into effective, targeted therapeutics. Collectively, this work highlights the potential of human iPSC derived-NVs to activate protective and regenerative mechanisms in the context of IRI, including at a cardiac cell type specific level. Crucially, we extend our study beyond solely a description of the expression of these proteins by also demonstrating the functionality and relevance of protein networks in our MT model system to IRI in cardiac injury response. We demonstrate that while CERA NVs primarily acts *via* direct cytoprotective signaling in parenchymal CMs, CL2 NVs operates predominantly as a potent suppressor of non-myocyte activation and ECM remodeling following ischemic injury.

Cardiac repair following MI remains a challenge in the clinic. Recent research has identified the need of a scalable multimodal approach given that the injury triggers simultaneously diverging signals known as cardiac remodeling. However, the study of the human heart represents a major hurdle to develop efficient therapies (105, 106). Engineered hCMT now represent mature, multicellular, human-relevant models that recapitulate structural integration, vascular-stromal crosstalk, and clinically meaningful functional responses under stress conditions such as IRI (107). Recent work in this field spans triculture heart-on-a-chip platforms with iPSC-derived CMs, fibroblasts, and endothelial cells that exhibit flow-responsive endothelium and catecholamine responses (107); hCMTs that support electrical pacing, arrhythmia simulation, and mitophagy reporters for IRI studies (108); and hCMTs that model acute MI and fibrotic remodeling under hypoxia-reoxygenation (3). Collectively, these advances underscore that human MTs can now reproduce hallmarks of IRI (contractile dysfunction, biomarker release, mitochondrial stress, and maladaptive ECM responses) while enabling high-content, mechanistic analyses, and therapeutic testing (3, 107, 108, 109, 110, 111).

Our study extends this trajectory by challenging iPSC-derived NVs in a human iPSC MT model of IRI (16, 37). The hCMT model used, as shown by Yin *et al*. (12), recapitulates key structural and functional features of human cardiac tissue, including the integration of CMs, fibroblasts, and endothelial cells and establishment of a communication network. We also demonstrate that in the absence of treatment, IRI induced marked contractile abnormalities such as prolonged contraction duration and time-to-peak, slowed relaxation, and a diminished relaxation/contraction velocity ratio that were accompanied by depressed beat-to-beat variability, a clinically used proxy of autonomic vagal/parasympathetic regulation in post-MI assessment (112). These changes mirror clinical observations that HRV declines after MI and correlates with recovery and risk, supporting the translational relevance of our readouts (112, 113, 114, 115, 116). Biochemically, hCMT subjected to IRI MTs exhibited a significant increase in cTnT release, consistent with the established role of cTns as biomarkers of CM injury and necrosis in MI (117, 118).

Our proteomics findings delineate a stress-response reprogramming under IRI. Control MTs were enriched in proteins associated with cardiac structure and function, including sarcomeric components and actin-binding proteins, reflecting a homeostatic myocardial phenotype. In contrast, IRI MTs exhibited upregulation of proteins involved in ER stress, unfolded protein response, and oxidative metabolism, including HYOU1 (62), HSPA5 (63, 64), and AIFM1 (69, 119), hallmarks of cellular stress and apoptosis. These findings are canonical molecular signatures of IRI pathobiology: ER stress sensors drive translational arrest, chaperone induction, and, when unresolved, apoptotic cascades (120); the integrated stress response converges on eIF2α phosphorylation to modulate translation during ischemia (121); and mitochondrial damage with impaired respiration and oxidative stress is central to reperfusion injury (122). The concordance of our proteome and functional assessments with these mechanisms and previous reports (70, 71, 123) validates the MT platform as a human surrogate for myocardial IRI.

Ideally for clinical utility under MI, a cardiac repair therapy will regulate simultaneously cell survival, angiogenesis, and scar formation. In this study, we show that following hypoxia a single dose of either iPSC NVs (CL2 or CERA) administered during reperfusion, increased the expression of prosurvival factors (G3BP2, GPD2, RPPRC, MDH2, and NIPSNAP1) (75, 76, 77, 78, 79) on the treated MTs. This indicates preservation of cellularity count in the treated groups and further confirmed as a reduction of CM cell death measured as free-cTnI (*p =* 0.005). These results extend previous findings in 2D cultures and underscore the therapeutic potential of iPSC-derived NVs in myocardial injury (34).

We show that NVs treatment ablate the disfunction induced by IRI in MT contractility by recovering basal levels of contraction duration, time to peak, relaxation time, and the ratio of relaxation to contraction velocities which aligns with the increase in the expression of proteins associated with cardiac function and contraction (CTNNA1, MYH6, NUP205, PIP4K2C, SLMAP, SIRP1, and SORBS2) (89, 90, 91, 92, 93, 94, 95) as revealed by mass spectrometry analysis. Furthermore, and in line with a multimodal approach for cardiac tissue repair, our proteomic analysis shows that NVs induce the expression of proteins associated with promotion of angiogenesis (ECE1 and FARSA) (73, 74) while also modulating fibrosis regulators (ITGB3) (96). Further functional analysis associated to these mechanisms is warranted. In addition, our proteomic reprograming analysis also shows that NVs recover the homeostatic levels of proteins associated with ER-stress (HSPA9, NOMO2, PSMD5, RAB32, and STX5) (80, 81, 82, 83, 84) and mitochondrial dysfunction (EIF5, HADH, NDUFS2, PGP, and VDAC1) (85, 86, 87, 88). These processes have been shown to be hallmark to contend ischemia/reperfusion-induced damage. We propose that their regulation by NVs is key to preserve cellularity and function of the injured tissue. Further experiments to elucidate the mechanism and effect *in vivo* are warranted.

Interestingly, the proteomic signatures of NV-treated MTs differed depending on the source of iPSC. Both CL2 and CERA are well-known somatic iPSC lines used in cardiac differentiation studies. CL2 iPSC (39) were generated from human neonatal foreskin fibroblasts using viral transfection to mediate cellular reprogramming, whereas CERA iPSCs (40) were produced *via* a specific non-integrating reprogramming method. Here, CL2 NVs were potent in their remodeling function, and promoted enrichment in pathways associated with cardiac development, myofibril assembly, and contractile function, suggesting a reprogramming toward restoration of baseline cardiac physiology. In contrast, CERA NVs favored pathways related to mitochondrial integrity, cellular stress response, and membrane dynamics, indicating a more adaptive response to injury. This divergence and their background origin/origin reprogramming strategy, highlights their distinct functional utility towards tailoring NV-based therapies based on desired reparative outcome, and potential therapeutic use.

In summary, these results demonstrate that NVs mediate select, potent effect in CMT platform during reoxygenation that is sufficient to preserve contractility, promote CM survival, and reprogram their proteome toward cardiac repair and stress resilience. Crucially, our GSEA and rank-based proteomic analysis reveals that this cardiac remodeling is highly selective and strictly dependent on the cell origin of the NVs. While CERA NVs primarily drive direct parenchymal cytoprotection—uniquely upregulating critical structural and heat shock proteins (*Myh6*, *Des*, *Clu*, and *Hspb1/6/7*) within CMs—CL2 NVs function predominantly as potent microenvironmental regulators, delivering a targeted, comprehensive suppression of non-myocyte activation and fibrotic driving machinery (*Col1a2*, *Col3a1*, *Serpine2*, and *Fbn1*) within the fibroblast and endothelial compartments. Ultimately, these findings position iPSC-derived NVs as highly tunable, promising candidates for regenerative therapies, and establish cardiac MT platforms as powerful tools to screen and fine-tune cell-source-dependent NV selectivities for targeted IRI therapeutics (124).

## Data Availability

Data generated or analyzed during this study are included in this published article (and its supplementary information files) or available from Data Repositories. MS-based proteomics data are deposited to the ProteomeXchange Consortium *via* the MassIVE partner repository and available *via* MassIVE with identifier MSV000101964. Functional enrichment annotations were retrieved using g:Profiler (https://biit.cs.ut.ee/gprofiler/). Hierarchical clustering was performed in R and Perseus using Euclidian distance and average linkage clustering, with missing values imputed at z-score 0. R was also used for data visualization.

## Supplemental data

This article contains supplemental data.

## Conflict of Interest

The authors declare no competing interests.

## Figures and Tables

**Supplemental Figure:**