# Supplementary material for: An Integrated Cardiac Microtissue Proteome Map Extends Therapeutic Remodeling by Nanovesicles
Source: Mol Cell Proteomics. 2026 Aug 20;25(9):101643. doi: 10.1016/j.mcpro.2026.101643 (PMC13602716; doi:10.1016/j.mcpro.2026.101643)
Supplement: Supplementary file 1 [file mmc1.docx]

**Supplementary Figure 1. Human cardiac microtissue hypoxia/reoxygenation model.** (**B**) Representative immunofluorescence imaging of human cardiac microtissues: Normoxia and hypoxia/reoxygenation conditions. Scale 500 nm. DAPI, VIM (Vimentin), cTnT (cardiac Troponin).

**Supplementary Table 1 -** Proteome IDs of human cardiac organoids: Normoxia UT, IRI UT, CL2 NVs T and CERA NVs T - Log2 LFQ intensity

**Supplementary Table 2** - Imputed, normalised human cardiac organoids proteome

**Supplementary Table 3** - Z-scored statistically significant proteins between organoid groups.

**Supplementary Table 4** - Z-scored statistically significant proteins between Normoxia UT and IRI UT.

**Supplementary Table 5** - Z-scored statistically significant proteins between IRI UT, CL2 NVs T and CERA NVs T.

**Supplementary Table 6** - Z-scored statistically significant proteins between IRI UT and CL2 NVs T.

**Supplementary Table 7** - Z-scored statistically significant proteins between IRI UT and CERA NVs T.

**Supplementary Table 8** - Z-scored statistically significant proteins between CL2 NVs T and CERA NVs T.

**Supplementary Table 9** - Gene Ontology enrichment analyses of organoid proteome based on IRI UT

**Supplementary Table 10** - REACTOME pathway enrichment analyses of organoid proteome (IRI UT vs Treatments)

**Supplementary Table 11** - Gene Ontology enrichment analyses of organoid proteome - IRI UT and CL2 NVs T.

**Supplementary Table 12** - Gene Ontology enrichment analyses of organoid proteome - IRI UT and CERA NVs T.

**Supplementary Table 13** - REACTOME pathway enrichment analyses of organoid proteome - IRI UT vs CERA NVs T.

**Supplementary Table 14** - Z-scored statistically significant proteins common in CL2 NVs T and CERA NVs T (Scatter plot).

**Supplementary Table 15** - Differential proteins between CL2 NVs T and CERA NVs T, relative to IRI

**Supplementary Table 16** - Cell type-specific enrichment clusters and ischemic cardiac cell assignment
